# Dynamic Field Assessment of Canopy Development and Periderm Maturation in Potato (*Solanum tuberosum* L.)

**DOI:** 10.3390/plants14172780

**Published:** 2025-09-05

**Authors:** Connor L. Buckley, Fransico Gonzalez-Tapia, Duroy A. Navarre, Jacob M. Blauer

**Affiliations:** 1 Potato Physiology Laboratory, Department of Horticulture, Washington State University, Pullman, WA 99164, USA; connor.buckley@wsu.edu; 2 USDA-ARS Forage Seed and Cereal Research Unit, Prosser, WA 99350, USA; paco.gonzalez@usda.gov; 3 USDA-ARS Tree Fruit and Vegetable Research Unit, Prosser, WA 99350, USA; roy.navarre@usda.gov

**Keywords:** potato, canopy development, periderm maturation, phellem, skin set, shear-strength, tuber quality

## Abstract

Background: Periderm development in potato (*Solanum tuberosum* L.) is critical for protecting tubers from biotic and abiotic stress, yet the relationship between periderm maturation, canopy development, and tuber growth during the active growing season remains poorly understood. We hypothesized that cultivar-specific differences in canopy growth and biomass partitioning would influence the timing and extent of periderm structural development and tuber growth in field conditions. This study aimed to fill this gap by examining how periderm maturation aligns with canopy development and tuber growth in field conditions. Methods: Three commercial cultivars: Alturas, Clearwater Russet, and Russet Burbank, were evaluated in replicated field trials. Canopy biomass, tuber yield, and total biomass were measured at multiple stages, while periderm anatomy was assessed using histological imaging, and strength was quantified through shear force resistance from tuber initiation to vine kill. Results: Alturas exhibited the highest canopy biomass, tuber yield, and periderm strength. Phellem structure, measured by cell layer number and thickness, stabilized by approximately 100 days after planting, yet shear strength continued to increase until vine kill. Cultivar-specific differences were observed in the timing and extent of periderm maturation. Conclusions: Periderm strength at 50% harvest index provided a meaningful benchmark for evaluating skin set in early-harvest systems. These findings support more informed decisions for cultivar selection, harvest timing, and postharvest handling to reduce skinning damage and improve tuber quality.

## 1. Introduction

Potato (*Solanum tuberosum* L.) is a globally important crop, valued for its high productivity, nutritional content, and versatility across fresh and processed markets [1,2]. Maximizing potato yield requires a thorough understanding of canopy growth and its role in assimilate production and partitioning, while optimizing tuber quality depends, in part, on the development and maturation of the periderm (skin). The interaction between canopy dynamics and tuber development is a vital component of potato production, influenced by cultivar, environment, and management practices [3]. Biomass accumulation and partitioning between vegetative and reproductive growth are key determinants of tuber development and yield [4,5]. However, vegetative growth can elevate disease risk and create competition for assimilates, potentially reducing tuber yield and quality [6]. Understanding the balance between canopy biomass and tuber development is essential for optimizing potato production, especially as it relates to periderm development.

Physical characteristics of potato tubers, such as storage parenchyma, starch content, and periderm development, are central to both quality and storage shelf-life [7]. As potato tubers enlarge, the periderm must remain pliable to accommodate cell expansion while simultaneously providing protection to the parenchyma from environmental stressors. Additionally, anatomical features such as periderm cell size and structure can influence the susceptibility of tubers to mechanical damage and pathogen invasion during post-harvest handling [8]. Disruptions in this process may result in both biological infection and physiological disorders such as periderm disorder syndrome [9].

The native periderm initially forms from the hypodermis beneath the epidermis, developing into three specialized layers: the phelloderm (storage), phellogen (meristematic), and phellem (protective) [10,11]. The phelloderm provides carbohydrates to the phellogen, while the phellem (composed of suberized cells) serves as the primary barrier against biotic and abiotic stressors [12]. As tubers expand, new phellem cells are produced to accommodate surface growth and replace sloughed cells. Once formed, phellem cells continue to mature and produce suberin. Suberin is a lipid-phenolic polymer composed of a polyphenolic domain (SPP) within the cell wall and a polyaliphatic domain (SPA) deposited in the middle lamella [13]. These domains help regulate gas and water exchange, contribute to resistance against pathogen infection, and can be visualized as autofluorescence under UV light [14,15].

At the end of the growing season, typically at vine kill, tuber swelling ceases and the periderm undergoes maturation. This maturation involves additional suberin deposition in the phellem and strengthening of the phellogen cell walls to reinforce adhesion to the underlying phelloderm [16,17,18]. If harvest occurs prematurely or under mechanical stress, phellem may detach from the phelloderm along the ‘shear layer’, resulting in skinning injury. The force required to induce this detachment is defined as the ‘shear force’ [19]. Although numerous studies have examined periderm strength during and after maturation [12,16,20,21,22], relatively little is known about how periderm mechanical strength progresses during the active stages of tuber growth.

In the Columbia Basin of Washington State (USA), the growing season for commercial potato production can range from ~90 to nearly 200 days after planting (DAP), depending on cultivar and market class. Because the periderm must remain both flexible and protective throughout this period, understanding how canopy development influences the periderm structure and function is critical for minimizing mechanical damage, especially for early-harvest markets. This study evaluated three commercially important processing cultivars (Alturas, Clearwater Russet, and Russet Burbank) that differ in canopy architecture and storage performance.

These cultivars were selected because of their importance in the U.S. processing markets and represent contrasting physiological and agronomic traits. Alturas is a late-maturing, high-yielding cultivar with large tubers and strong storage performance, commonly used for dehydrated products [23]. Clearwater Russet in an early-to mid-maturing cultivar valued for its uniform shape, and high fry quality [24]. Russet Burbank, one of the most widely grown processing cultivars worldwide, is characterized by having a long growth cycle, high fry quality, and variable tuber set, but has lower yield potential and higher susceptibility to skinning injury compared to newer cultivars [25].

This research is one of the first to characterize periderm shear strength dynamically across the full growing season while simultaneously monitoring canopy growth and biomass allocation. We examined cultivar-specific patterns in canopy biomass, tuber bulking, periderm structure, and mechanical resistance in-field to better understand the interaction between vegetative development and periderm strengthening. These findings have direct implications for optimizing harvest timing, reducing skinning damage, tailoring management strategies, and improving storage quality for both full-season and early-harvest systems, including baby potato markets.

## 2. Results

### 2.1. Foliar Development

**Figure 1:**
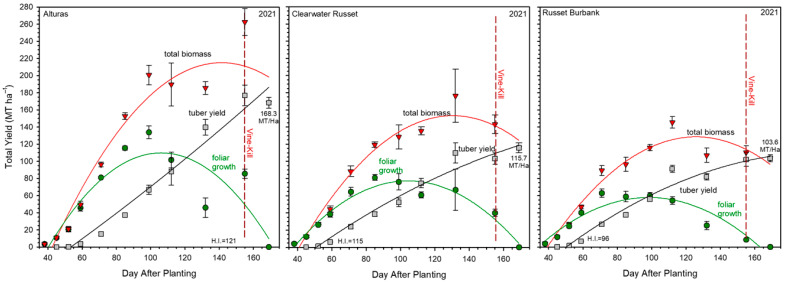
In-field growth profiles for cultivars, Alturas, Clearwater Russet, and Russet Burbank during the 2021 season for foliage growth (green circles), tuber development (gray squares), and the total biomass (red triangles; foliage + tuber yield). The days after planting (DAP) when the harvest index (H.I.) was equal to 50%, it was calculated from the quadratic trends for both the foliar and tuber yields. The corresponding DAP for the 50% HI provides insights into the contribution of canopy growth on tuber development. Error bars indicate standard error.

The foliar weight across the three cultivars showed differences at 155 DAP (*p* < 0.001; Figure 1, green circles). Throughout the season, foliage peaked at 99 days after planting (DAP) for Russet Burbank and 109 DAP for Alturas and 106 DAP for Clearwater Russet, as determined by quadratic regression analysis. At the foliage peak, Alturas was 42% greater than Clearwater Russet and 90% greater than Russet Burbank. The growth rate to peak canopy for Alturas was 1.04 metric tons (MT) ha^−1^ day^−1^, while Clearwater Russet was 0.74 MT ha^−1^ day^−1^ and Russet Burbank was 0.58 MT ha^−1^ day^−1^. The rate of foliar decay until vine kill at 155 DAP from peak foliar weight was calculated at −1.23 MT ha^−1^ day^−1^ for Alturas, −0.79 MT ha^−1^ day^−1^ for Russet Burbank, and −0.89 MT ha^−1^ day^−1^ for Clearwater Russet (Table 1). Among the three cultivars, Russet Burbank had the least foliage at vine kill (13.61 MT ha^−1^).

### 2.2. Tuber Development

Tuber total yield was recorded throughout development and bulking stages (Figure 1, gray squares). Total tuber yields increased until vine kill at 155 DAP, with the effect of cultivar impacting tuber yield (*p* < 0.01). At 169 DAP, Alturas produced 54% more tuber yield (168.30 MT ha^−1^) than Clearwater Russet (115.68 MT ha^−1^) and Russet Burbank (103.57 MT ha^−1^). On average, tuber yields increased at rates of 1.00 MT ha^−1^ day^−1^ for Alturas, 0.68 MT ha^−1^ day^−1^ for Clearwater Russet, and 0.61 MT ha^−1^ day^−1^ for Russet Burbank (Table 1). Tuber counts per plant were highly influenced by cultivar (*p* < 0.001). On average, at vine kill (155 DAP), Alturas had 19, Clearwater had 14, and Russet Burbank had 10 tubers per plant. The average specific tuber weight was also impacted by cultivar (*p* < 0.001) with Clearwater Russet producing the smallest tubers (163 g) at vine kill and Alturas and Russet Burbank producing equivalent sized tubers (196 g and 214 g, respectively).

### 2.3. Total Biomass

The average total biomass (calculated as the sum of foliar weight and tuber yield) differed among cultivars (*p* < 0.001; Figure 1; red triangles). At vine kill (169 DAP), Alturas produced the highest total biomass (262.47 MT ha^−1^), significantly exceeding Clearwater Russet (176.35 MT ha^−1^), which in turn surpassed Russet Burbank (145.39 MT ha^−1^). Using a quadratic equation, we estimated the day after planting (DAP) at which the harvest index (HI) reached 50% (the point when tuber yield equaled foliar yield) as an indicator of when cultivars shifted allocation from canopy growth to tuber development. Russet Burbank reached this threshold the earliest at 96 DAP, followed by Clearwater Russet at 115 DAP and Alturas at 121 DAP.

After the point when HI reached 50%, Alturas took the most DAP to reach peak total biomass at 140 DAP, reaching an estimated 215.06 MT ha^−1^. This corresponded to the highest calculated daily biomass accumulation rate among cultivars at 1.54 MT ha^−1^ day^−1^ (Table 1). Due to continued late-season foliar regrowth and tuber development, Alturas accumulated biomass up to vine kill at 155 DAP, preventing a reliable estimate of post-peak total biomass decline.

Clearwater Russet reached peak biomass at 130 DAP with an estimated 152.82 MT ha^−1^, accumulating biomass at a rate of 1.18 MT ha^−1^ day^−1^. After reaching its peak, biomass declined steadily over the following 25 days to 143.04 MT ha^−1^ at vine kill (155 DAP), representing a loss rate of −0.39 MT ha^−1^ day^−1^ (Table 1).

Russet Burbank showed the earliest DAP to reach peak biomass at 126 DAP, reaching 128.13 MT ha^−1^ with a daily accumulation rate of 1.02 MT ha^−1^ day^−1^. Biomass declined thereafter at a rate of −0.61 MT ha^−1^ day^−1^, reaching 110.35 MT ha^−1^ at vine kill (155 DAP) (Table 1). Overall, the timing of peak biomass followed the order: Alturas (latest), Clearwater Russet (intermediate), and Russet Burbank (earliest), reflecting underlying differences in canopy persistence and growth duration among cultivars that aligned with observations of DAP for a 50% HI.

### 2.4. Shear Resistance

**Figure 2:**
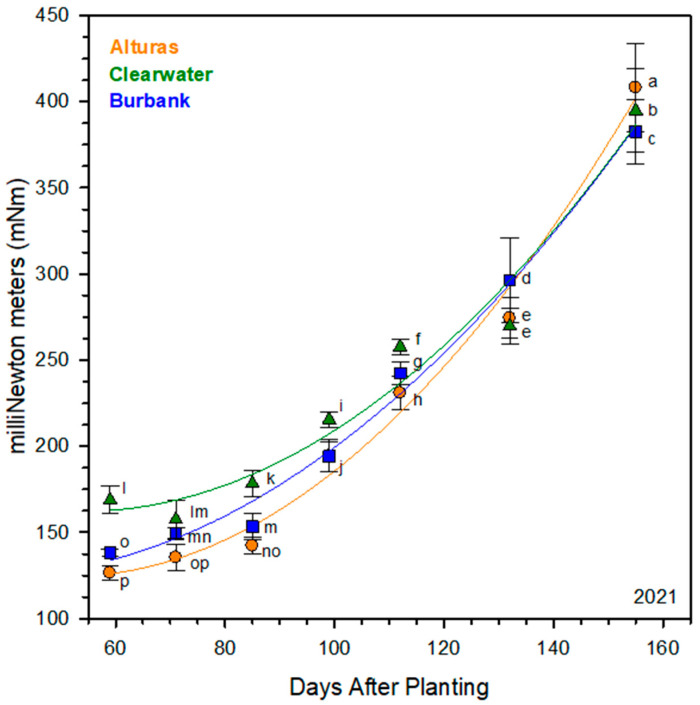
Torque required to shear the periderm from the tuber cortex measured in milliNewton meters (mNm) for each hand dig date in 2021. The force required increased from 59 to 155 days after planting (DAP). The increase in shear resistance is estimated for each cultivar using a quadratic trendline. Error bars represent the standard error for each data point and statistical significance is demonstrated using connecting letters (*p* < 0.05).

The shear resistance of all cultivars significantly increased throughout the growing season. A notable difference in shear resistance was found among the cultivars, particularly during the first 99 DAP of tuber development (*p* < 0.05; Figure 2). The average shear resistance from 59 to 99 DAP was the highest for Clearwater Russet at 179.47 mNm, Russet Burbank at 158.68 mNm, and Alturas with a shear resistance of 149.61 mNm.

The shear resistance was significantly different between all three cultivars for both the average (over the growing season) and at vine kill (155 DAP), though the differences between the cultivars depended on if the average value or the final value was considered. On average, Clearwater Russet had the greatest shear resistance (234.30 mNm), followed by Russet Burbank (222.30 mNm), and Alturas (215.94 mNm). Alternatively, at only 155 DAP, Alturas had the greatest shear resistance (408.12 mNm), followed by Clearwater Russet (394.84 mNm), and Russet Burbank (382.63 mNm). This observation at 155 DAP aligns with the quadratic regression analysis, where Alturas initially exhibits lower skin shear resistance compared to the other two cultivars, but by approximately 145 DAP, it surpassed both. All three cultivars showed progressive increases in shear resistance as the season progressed.

### 2.5. Phellem Cell Count

**Figure 3:**
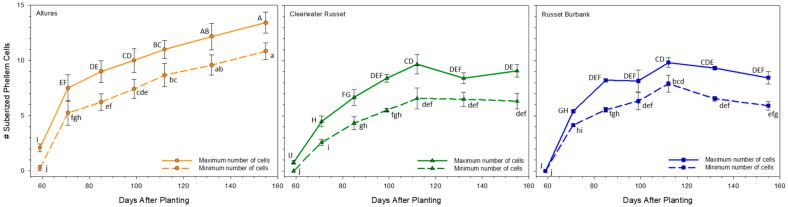
Histological sampling was completed for each hand dig date during the 2021 growing season. Samples were hand-sectioned to evaluate the increasing number of phellem cells (layers thick) throughout the growing season. Three photos were taken from each replication, and the phellem layers with the greatest and least number of suberized cells were counted and recorded. Error bars represent the standard error and statistical significance is demonstrated using connecting letters (*p* < 0.05). Lower case letter denotes statistics for the average minimum number of cells counted and upper-case letter denotes statistics for the average maximum number of cells counted.

**Figure 4:**
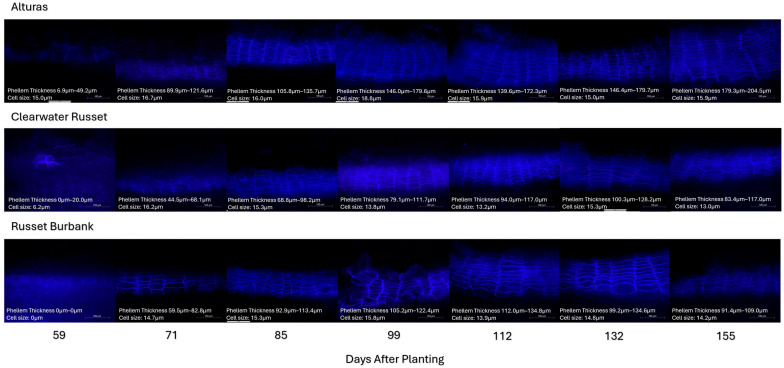
UV autofluorescence light microscopy was used to quantify the development of the phellem throughout the 2021 growing season. The suberin polyphenolic domain deposited between the phellem cells is visible under UV light and allows for the quantification of mature phellem cells. The phellem layer with the greatest and the least number of cells was found on each usable photo. The thickness of both layers was measured using ImageJ. This was repeated three times for each replication and a representative photo for each sampling date is shown. Cell size is the phellem layer thickness (average) divided by the average number of cells in the phellem layer to give the average cell size. This was not directly measured but rather calculated.

The minimum and maximum number of phellem cells were counted for each cultivar and for each sample date by counting the number of visible cells that fluoresced under UV light (Figure 3 and Figure 4). Overall, the minimum and maximum phellem cell numbers were impacted by both the cultivar and days after planting (*p* < 0.001), but the interaction was only observed for the minimum number of cells (*p* < 0.05). At approximately 100 DAP, the average minimum and maximum number of cells (across cultivars) gained by the tubers slowed, and there were no differences in the cell counts after 112 DAP through vine kill (Figure 3). When individual cultivars were averaged over the whole growth period, the minimum number of cells were different for each cultivar (*p* < 0.05); Alturas (ca. 6.9), Russet Burbank (ca. 5.2), and Clearwater Russet (ca. 4.6). When compared for each sample date, the minimum number of phellem cells was equivalent for Clearwater Russet and Russet Burbank, but after 71 DAP and beyond, the minimum number of cells that Alturas produced were more than Clearwater Russet. Alturas also began producing more cells than Russet Burbank after 132 DAP until vine kill. At vine kill, the minimum number of cells Alturas produced was 10.9, Clearwater Russet was 6.3, and Russet Burbank was 5.9 (Figure 3; dashed lines).

Similarly to the minimum number of cells, the maximum number of phellem cells was equivalent for Clearwater Russet and Russet Burbank across the growing season. When compared to Alturas, there were also similar trends, but slight observable differences. For Alturas, the maximum cell count was higher than Russet Burbank at 59 and 71 DAP, and higher than Clearwater Russet at 71 and 85 DAP (*p* < 0.05). The maximum cell count was then equivalent across all three cultivars until 132 DAP, at which point, the maximum number of cells in Alturas was again more than Clearwater Russet and Russet Burbank (*p* < 0.05). Both Clearwater Russet and Russet Burbank were equivalent for each sample date and when averaged over the whole season. The difference in cell counts between Alturas and the other two cultivars remained consistent from 132 DAP until vine kill at 155 DAP. At vine kill, the maximum number of cells for Alturas was 13.4, Clearwater Russet was 9.1, and Russet Burbank was 8.5 (Figure 3; solid lines).

### 2.6. Phellem Thickness

**Figure 5:**
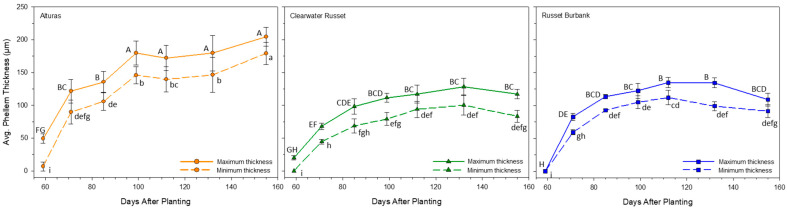
Histological sampling was completed for each hand dig date during the 2021 growing season. Samples were hand-sectioned to evaluate the phellem total thickness throughout the growing season. Phellem cell thickness measurements were made on areas of the phellem that had previously been identified as having the greatest and least number of phellem cells (Figure 3). The maximum thickness was recorded at the point of the phellem with the greatest number of layers. The minimum thickness was recorded at the point of the phellem where the least number of layers was identified. Error bars represent the standard error and statistical significance is demonstrated using connecting letters (*p* < 0.05). Lower case letter denotes statistics for the average minimum thickness and upper-case letter denotes statistics for the average maximum thickness.

Both the minimum and maximum phellem total thickness were measured under UV light during histological evaluation (Figure 4 and Figure 5). Phellem thickness (min. and max.) was impacted by both the cultivar and the DAP (*p* < 0.001), but only the interaction for the minimum phellem thickness was significant (*p* < 0.05). The minimum and maximum phellem thickness was equivalent for all three cultivars until 71 DAP. After 71 DAP, the minimum thickness was greater in Alturas than in Clearwater Russet and remained greater until vine kill at 155 DAP (Figure 5; dashed lines). Alturas was consistently greater than Russet Burbank after 132 DAP. Clearwater Russet and Russet Burbank were equivalent the entire season. At vine kill, the minimum thickness for Alturas was approximately two times that of Clearwater Russet (83.4 µm) and Russet Burbank (91.3 µm) at 179.2 µm.

Similarly, Alturas consistently produced the greatest maximum thickness at 99 DAP until 155 DAP (vine kill), but there were no differences between Clearwater Russet and Russet Burbank. Overall, Alturas again had approximately two times the maximum thickness compared to Clearwater Russet (117.0 µm) and Russet Burbank (109.0 µm) at 204.5 µm. The difference between the minimum and maximum phellem thickness for each cultivar at vine kill (155 DAP) was 25.3 µm (Alturas), 33.6 µm (Clearwater Russet), and 17.7 µm (Russet Burbank), demonstrating that phellem thickness varied the most for Clearwater Russet.

## 3. Discussion

### 3.1. Canopy and Tuber Development in Relation to Biomass Partitioning

Alturas exhibited the most vigorous canopy development, peaking at 106 days after planting (DAP), which supported the greatest total biomass accumulation (262.47 MT ha^−1^) and tuber yield (168.30 MT ha^−1^). These results are consistent with earlier studies showing that expansive canopy development enhances photosynthetic capacity, leading to increased biomass production and tuber yield [4].

In contrast, Russet Burbank had the lowest total biomass accumulation for both foliage and tubers. However, its tuber-to-foliage mass ratio (1.79) exceeded that of Alturas (1.53) and Clearwater Russet (1.50), suggesting a greater tuberization potential relative to canopy size. This distinction emphasizes partitioning efficiency, not just total biomass, as a determinant of productivity. The persistent foliage present at the time of vine kill (155 DAP) in Alturas and Clearwater Russet likely diverted assimilates to vegetative maintenance, underscoring the trade-off between prolonged canopy duration and efficient tuber bulking. This finding aligns with observations that excessive vegetative growth can also negatively impact yield by increasing competition for assimilates and elevating susceptibility to disease [26,27], though disease was not an evaluation metric in this study.

The timing of canopy senescence relative to tuber development further highlights cultivar differences. Russet Burbank reflects a more determinate growth habit, transitioning to tuber bulking earlier, while Alturas delays this transition, allowing for a larger canopy. Although this prolonged canopy activity supported higher yields in Alturas, its benefit depends on whether late-season foliage remains photosynthetically productive. When photosynthesis is maintained, extended canopy duration enhances tuber bulking; when it declines, vegetative persistence only increases maintenance cost or disease risk [28].

From a breeding perspective, these contrasting strategies highlight complementary opportunities. Cultivars senescing earlier, like Russet Burbank, may be better suited to short-season or stressed environments where rapid partitioning efficiency is prioritized. In contrast, late-senescing cultivars such as Alturas can achieve superior yields in longer growing seasons if the canopy remains photosynthetically productive. Balancing canopy duration with tuber bulking capacity is therefore a central challenge for optimizing both yield and resilience.

Alturas also demonstrated a greater capacity to support tuber set, producing nearly twice as many tubers per plant as Russet Burbank while maintaining a similar average size tuber. This capacity reflects not just canopy persistence but greater photosynthetic productivity per unit of biomass. The extended time to reach 50% HI in Alturas allowed for greater leaf area development, which in turn supported prolonged assimilate partitioning to tubers which resulted in greater tuber yield. This observation supports the concept that a longer vegetative growth period can enhance tuber yield by increasing assimilate supply [29].

Together, these cultivar-specific differences in canopy dynamics and partitioning efficiency highlight important trade-offs in breeding and management. While Russet Burbank exemplifies efficient tuberization relative to canopy size, Alturas illustrates the yield advantage granted by prolonged canopy assimilation. Optimizing these traits across production environments will require balancing efficiency, canopy persistence, and disease resistance to meet both yield and quality goals. Furthermore, the cultivar-specific patterns in tuber development have direct implications for tuber bulking rate and capacity, both of which depend on continued tuber growth. This growth, in turn, requires an active periderm to protect and support the expanding tuber.

### 3.2. Periderm Development and Shear Strength

Periderm strength plays a key role in minimizing mechanical damage and supporting postharvest quality. Previous research has focused extensively on the anatomical development and genetic regulation of the periderm [10,11,12,14,30,31]. However, studies have not evaluated the dynamic mechanical strength of periderm during active tuber bulking. Most existing work characterizes skin strength post-vine kill or during storage [12,16,20,21,32,33], missing the critical development window when periderm is laid down during rapid tuber expansion. Given that potatoes are harvested at varying tuber sizes, depending on the end-use (baby potatoes, baker potatoes, fries, chips, seed, etc.), it is important to understand the impact of tuber size on periderm strength to reduce skinning damage.

Kumar and Ginzberg [8] emphasize that periderm development, particularly suberization and the formation of the phellem cell layer, is essential for minimizing mechanical injury and reducing the risk of pathogen entry during harvest and storage. Lulai [34] clarified that skinning results from two structural failures: (1) fracturing of the phellem cell walls, and (2) fracturing of the phellogen cell walls, also known as the ‘phellogen shear component’. During active tuber bulking, the periderm must remain sufficiently flexible to accommodate expansion, requiring continuous production of new phellem cells to cover the enlarging parenchyma. This balance between flexibility and strength has led to the assumption that periderm structure, and therefore its resistance to mechanical damage, remains relatively weak until vine kill, when tuber growth ceases and cell walls begin to thicken.

Our data supports and extends this model. Specifically, we show that (1) periderm structure (number of phellem cells and phellem total thickness/size; Figure 3 and Figure 5) is largely established by approximately 100 DAP, (2) the periderm strength increases with a prolonged growing period (Figure 1), and (3) periderm quality is dependent on the genotype, even before vine kill (Figure 2, Figure 3, Figure 4 and Figure 5). These findings indicate that functional strengthening of the periderm occurs later than structural development and is not necessarily reflected in phellem layer thickness or cell counts. Rather, the increase in resistance likely arises from biochemical processes, including the deposition of suberin and pectin cross-linking, that reinforce the periderm mechanically over time [14,16]. Our findings suggest that anatomical stability of the periderm precedes biochemical reinforcement, which continues until vine kill. For breeding and management, this distinction is critical: cultivars that achieve earlier periderm strengthening may be better suited for early harvest markets, while those requiring a longer maturation period may be provided advantages for storage quality. Integrating periderm strength assessments into cultivar evaluation could therefore improve both harvest management and postharvest handling practices.

Alturas consistently exhibited the thickest phellem layer (204.5 µm compared to 117.0 µm in Clearwater Russet and 109.0 µm in Russet Burbank) and demonstrated the highest shear resistance at vine kill (408.1 mNm compared to 394.8 mNm in Clearwater Russet and 382.6 mNm in Russet Burbank). These traits are indicative of enhanced suberin biosynthesis, which contributes to both the protective barrier function and mechanical strength of the periderm [13,35] and provides a cultivar-specific advantage for early skin set and improved postharvest handling.

### 3.3. Timing of Skin Set and Cultivar-Specific Implications

When shear strength values were aligned to each cultivar’s 50% HI, Alturas still outperformed Russet Burbank (248 mNm vs. 190 mNm), while values for Alturas and Clearwater Russet were similar (248 mNm vs. 245 mNm; Figure 2). This suggests that although Russet Burbank has an earlier physiological transition from vegetative to reproductive growth, its periderm does not strengthen as robustly or rapidly as that of Alturas. This has important implications for markets requiring early harvest (e.g., baby potatoes), where strong periderm formation must occur under shorter growth windows. Cultivar-specific timing of canopy maturity and periderm strengthening should therefore be considered when targeting early-harvest systems.

Interestingly, despite differences in tuber sizes among and between cultivars, average phellem cell size and phellem thickness (Figure 3, Figure 4 and Figure 5) remained consistent after approximately 100 DAP. This suggests that expansion of the periderm surface area in larger tubers results primarily from increased cell proliferation rather than cell enlargement. Phellem cell layer counts in Alturas increased until 132 DAP, but cell dimensions and phellem layer thickness remained stable beyond 100 DAP, further supporting this hypothesis. Strength gains after 100 DAP are thus likely due to progressive reinforcement of the phellogen cell walls rather than gross structural remodeling of the outer layers, and this strengthening occurs before vine kill.

Our anatomical (Figure 4) and shear strength assessments (Figure 2) focused on the equatorial region of the tuber, though differences in developmental activity between the stem and bud ends may explain some variability. Past research has shown that at harvest, dry matter content is higher in the stem end compared to the bud end. Gene expression studies further clarified that the bud end has higher starch synthesis gene expression and lower sucrose transport gene expression compared to the stem end of the tuber, which indicates most tuber growth at harvest is occurring at the bud end of the tuber [36]. Our observations demonstrate a lack of change in periderm cell counts and phellem thickness, which may align with an anatomically developed periderm at the equatorial region of the tuber at 100 DAP, whereas continued structural development may occur at the bud up until vine kill. Continued swelling at the equatorial region is therefore protected by cell division at a lower rate than that required at rapidly expanding sites such as the bud end of the tuber. Implications for these observations suggest that skinning damage is likely greater at the bud end of the tubers compared to the stem end and while maturation practices improve periderm strength generally, the bud of the tuber benefits the most.

## 4. Materials and Methods

### 4.1. Plant Material and Field Conditions

Certified G3 seed potatoes (*Solanum tuberosum* L.; cvs. Alturas, Russet Burbank, and Clearwater Russet) were sourced from a commercial seed grower in Reardon, WA, in January 2021. Tubers were stored at the Washington State University potato storage at 4 °C (95% + RH) until cutting and planting in April 2021. The seed tubers were cut by hand into 50–64 g seed pieces and sorted for apical and basal portions (stem and bud). Equal amounts for each portion were used in the field study. Seed tubers were allowed to wound-heal at 12 °C (95% + RH) for three days before planting.

The seed was planted at a depth of 20.3 cm (25.4 cm in-row spacing and 81.3 cm between-row spacing) in a Shano silt loam soil [37] at the Washington State University Irrigated Research and Extension Unit at Othello, WA (46°47′35.4″ N. Lat., 119°02′19.3″ W. Long.) using a custom two-row carousel hand-feed planter. Two field trials were planted in tandem, one to support hand-dig assessments before vine kill and the second to assess total tuber yield post-vine kill. Plots for hand-dig sampling were arranged in a randomized complete block design with four field replicates, and plots for the total yield (post-vine kill) included five field replicates. Individual plots consisted of 8 or 24 plants (hand-dig or total yield trials, respectively), including a single red potato plant (cv. Chieftain) at the beginning and end of each plot to facilitate plot separation. Each plot was flanked with Russet Burbank to promote plant competition. The crop was cultivated according to standard practices for long-season russets in the Columbia Basin of Washington [38] and allowed to grow up to 155 days before termination of hand-dig sampling or before vine removal using a mechanical flail mower (vine kill) for the total yield trial. A total of 10 hand-digs occurred during the growing season with sampling at 38, 45, 52, 59, 71, 85, 99, 112, 132, and 155 days after planting (DAP). Harvest of the total yield trail was accomplished using a single-row bagger harvest (Braco^®^) 14 days after vine kill for a total of 169 days from planting to harvest.

### 4.2. Phenotyping and Sampling

#### 4.2.1. Yield and Tuber Size

Whole plants were hand harvested from each plot on the corresponding harvest date. The total number of tubers were counted (smallest tubers were 2 times the diameter of the attached stolon or larger) and measured gravimetrically. Data was collected to determine the tuber weight, foliar weight, and total biomass weight. Individual tuber weights and counts were used to estimate the average tuber size for each sample and date (Figure 1 and Supplemental Figure S1). The final total yield was determined by harvesting each plot, and individual tubers were then weighed and counted using a custom potato sorter.

#### 4.2.2. Periderm Shear Strength

Starting at 59 DAP, the shear force strength to delaminate (shear) the periderm from the tuber cortex was measured following a modified protocol from Lulai et al. [19]. In short, 40 random, intact tubers were selected from each plot, and shear force strength was measured onetime at the equatorial region of each tuber (*n* = 160 tubers). Tubers were washed free of excess soil and surface air-dried before measuring. Shear force was measured using a square drive torque-measuring screwdriver (McMaster-Carr; product number 54765A11, Chicago, IL, USA; 0 to 192 in.oz. and 0 to 12 in.lbs. torque) with a custom tester tip (Mayo Manufacturing Inc., Grand Forks, MN, USA, product: Skin Tester Set Potato). The tester tip was composed of a #1 black rubber stopper (1.6 cm diameter on the narrow end) with 120-grit sandpaper attached to the rubber stopper head to improve contact with the tuber, a 2.6 N/mm compression spring to ensure consistent contact force against the tuber, and a flywheel to improve tool action when shearing occurred to prevent measurement error. After the periderm was sheared from the cortex, the force required was recorded from the torque-measuring screwdriver and converted to millinewton meter [mNm] units.

#### 4.2.3. Histological Sampling

Four average-sized tubers per field replication were cut in half longitudinally. One-half of each tuber was again sliced by hand to create slices ranging from 0.5 to 1 cm in width. Surplus cortex tissue was removed, leaving about 0.5 cm below the periderm of one side of the tuber slice, and a 3 to 4 cm equatorial tuber portion was saved for later histological sampling. The tuber portions were immediately preserved in 15 mL Falcon test tubes filled with a formaldehyde (3.7%), ethanol (50%), acetic acid (5%) fixative (FAA; [14]) to maintain cellular integrity.

After fixation, samples were stored at 4 °C until sectioning. Hand sections (0.3–0.1 mm thickness) were prepared immediately prior to imaging, stained using 0.02% ruthenium red for 10 min, rinsed with distilled water, and mounted with distilled water on glass slides. Autofluorescence of suberin polyphenolic domains was visualized using a Leitz Aristoplan Fluorescent Light Microscope, equipped with a D-filter cube (excitation: 350/50 nm, emission: 460/50 nm), at a magnification of 25× (Figure 4).

Histological periderm profiling focused on phellem development. A total of three hand sections/photos (technical replications at 25× magnification) per replication (*n* = 4 total replications) were used to assess the progress of suberization by counting the number of and measuring the phellem cells exhibiting autofluorescence under UV light, a characteristic conferred by the polyphenolic domain of suberin [14]. The minimum and maximum number of cells and phellem thickness were recorded using software ImageJ (version 1.53s bundled with Zulu OpenJDK 13.0.6) [39] across three hand sections per replication.

Average phellem cell size was calculated indirectly by dividing the total phellem layer thickness by the corresponding number of cells in that layer, as measured in imageJ (version 1.53s). This approach provided an estimate of mean cell size without direct measurement of individual cell diameters.

### 4.3. Data Analysis

To quantify the rate of change in foliar and tuber growth over time, the average ascent and descent rates were calculated using cultivar-specific quadratic models fitted to the observed data. Each curve was described by the following general equation:

$$f\left(x\right)=ax^{2}+bx+c$$

where a is the quadratic coefficient, b is the linear coefficient, and c is the y-intercept. The peak point (representing the maximum value of the trait) was first identified by solving for the DAP at which the first derivative of the quadratic function equaled zero. The ascent rate was then calculated as the average rate of increase in the trait between zero days after planting (DAP), based on the model, and the peak DAP, while the descent rate was calculated as the average rate of decline between the peak DAP and the final observation at 155 DAP (169 DAP for tuber yield). These were computed using the following formulas:

$$Ascent\;Rate=\;\frac{f\left(x_{peak}\right)-f(0)}{x_{peak}-0}$$

$$Descent\;Rate=\frac{f\left(155\right)-f(x_{peak})}{155-x_{peak}}$$

This approach provided a standardized framework to compare developmental patterns across cultivars and traits by capturing both the magnitude and timing of growth dynamics. The harvest index (HI) was calculated from the quadratic line equation for both the foliar and tuber yields and represents the DAP at which the HI is equal to 50% for each cultivar. The DAP value demonstrates the differences in foliar growth between the cultivars to identify canopy differences that support tuber development.

Observations of foliar growth, total biomass, tuber yield, and phellem cell number and thickness (*n* = 4) were analyzed using a one-way analysis of variance, while shear strength (*n* = 160) was analyzed using a two-way analysis of variance. Cultivar and DAP were treated as fixed effects, while the replication was included as a blocking effect among plots. Model assumptions, including normality of residuals and homogeneity of variance, were evaluated using residual plots and diagnostic tests. Post hoc comparisons were performed using Student’s *t*-test. Statistical significance was determined based on ANOVA followed by post hoc *t*-tests, with a threshold of *p* < 0.05. The analyses were conducted using JMP V15 (SAS Institute Inc., Cary, NC, USA, 1989–2025). At 85 DAP, one sample for Alturas and Russet Burbank were lost and replication values were calculated using the average of the remaining replications. Figures were developed using SigmaPlot 11.0 (Grafiti, LLC, Palo Alto, CA, USA). Calculations and tables were developed using Microsoft Excel (Microsoft 365).

## 5. Conclusions

While past studies have focused on anatomical and genetic regulation of periderm development during tuber bulking, research on periderm strength has been limited to tuber maturation (vine kill to harvest) and through storage. Because of this gap, this study investigated the concurrent development of canopy biomass, tuber bulking, and periderm structure and strength throughout the active growing season, from tuber initiation to vine kill, in three commercially important potato cultivars.

Our results highlight the influence of cultivar on canopy development, tuber yield, and periderm strength. Under our experimental conditions, Alturas generally exhibited higher values across these traits, but cultivar responses were not uniform, and cultivar performance may vary under different environments or management practices. Phellem thickness was largely established by approximately 100 days after planting, and the number of phellem cell layers varied by cultivar. Despite this structural plateau, periderm shear strength continued to increase through the remainder of the growing period, suggesting that strengthening is driven more by biochemical reinforcement than continued anatomical expansion. Notably, periderm strength measured at the 50% harvest index (HI) offered a useful physiological benchmark for evaluating cultivar performance under early-harvest conditions, such as baby potato production.

These findings provide new insights into the timing and coordination of canopy decline and periderm maturation, with implications for variety selection and harvest management. They also support the use of periderm strength profiling in breeding programs aimed at reducing skinning injury and improving postharvest quality. Future work should investigate variation in periderm strength between the stem and bud ends of the tuber, as it relates to skinning prevalence, and to elucidate genetic regulation controlling periderm development and strength.

## Figures and Tables

**Table 1:** Model parameters, describing the growth and decay rates for foliar, total biomass, and tuber development.

Observation	Cultivar	* bx^2^	* ax	* c	Peak DAP	Peak Value ^a^	Growth Rate ^b^	Decay Rate ^b^	R-Sq.
Foliar Weight	Alturas	−0.025	5.36	−175.03	106	109.87	1.04	−1.23	0.78
Foliar Weight	Clearwater Russet	−0.0174	3.64	−112.31	104	77.00	0.74	−0.79	0.94
Foliar Weight	Russet Burbank	−0.0138	2.72	−76.22	99	57.74	0.58	−0.89	0.87
Total Biomass	Alturas	−0.022	6.23	−225.05	140	215.06	1.54	-	0.92
Total Biomass	Clearwater Russet	−0.0191	4.99	−171.97	130	152.82	1.18	−0.39	0.96
Total Biomass	Russet Burbank	−0.0171	4.31	−142.93	126	128.13	1.02	−0.61	0.94
Tuber Weight	Alturas	0.0015	1.28	−72.75	169	186.4	1.00	-	0.97
Tuber Weight	Clearwater Russet	−0.0032	1.71	−78.85	169	118.54	0.68	-	0.97
Tuber Weight	Russet Burbank	−0.0049	1.96	−86.25	169	116.51	0.61	-	0.96

* bx^2^, ax, and c represent the quadratic coefficient, slope coefficient, and the y-intercept, respectively. ^a^ Peak value is calculated from the line equation and uses Peak DAP to estimate the yield in MT ha^−1^. ^b^ Growth and Decay rates are calculated from the line equation in MT ha^−1^.

## Data Availability

The original contributions presented in this study are included in the article. Data is available for download at www.potatoes.wsu.edu (accessed on 6 August 2025) or at https://s3.wp.wsu.edu/uploads/sites/2742/2025/09/Research-Data.xlsx. Further inquiries can be directed to the corresponding author.

## Supplementary Materials

The following supporting information can be downloaded at https://www.mdpi.com/article/10.3390/plants14172780/s1: Figure S1: Photos of tubers from hand-digs at 132 days after planting for each cultivar. (A) cultivar Alturas. (B) cultivar Russet Burbank. (C) cultivar Clearwater Russet.

## Abbreviations

The following abbreviations are used in this manuscript:

DAP	Days After Planting
HI	Harvest Index
UV	Ultra-violet
SPP	Polyphenolic Domain
SPA	Polyaliphatic Domain
USA	United States of America
WA	Washington State
WSU	Washington State University
USDA	United State Department of Agriculture
RH	Relative Humidity
in.oz.	Inch Ounces
in.lbs.	Inch Pounds
N	Newtons
mNm	Millinewton meter
FAA	Formaldehyde; Alcohol; Acetic Acid Fixative
CV	Cultivar
MT	Metric Ton
Ha	Hectare
cm	Centimeter
µm	Micrometer
