# Supplementary material for: Dynamic Field Assessment of Canopy Development and Periderm Maturation in Potato (Solanum tuberosum L.)
Source: Plants (Basel). 2025 Sep 5;14(17):2780. doi: 10.3390/plants14172780 (PMC12431376; doi:10.3390/plants14172780)
Supplement: Supplementary file 1 [file plants-14-02780-s001.zip › plants-3834342-supplementary.pptx]

## Slide 1
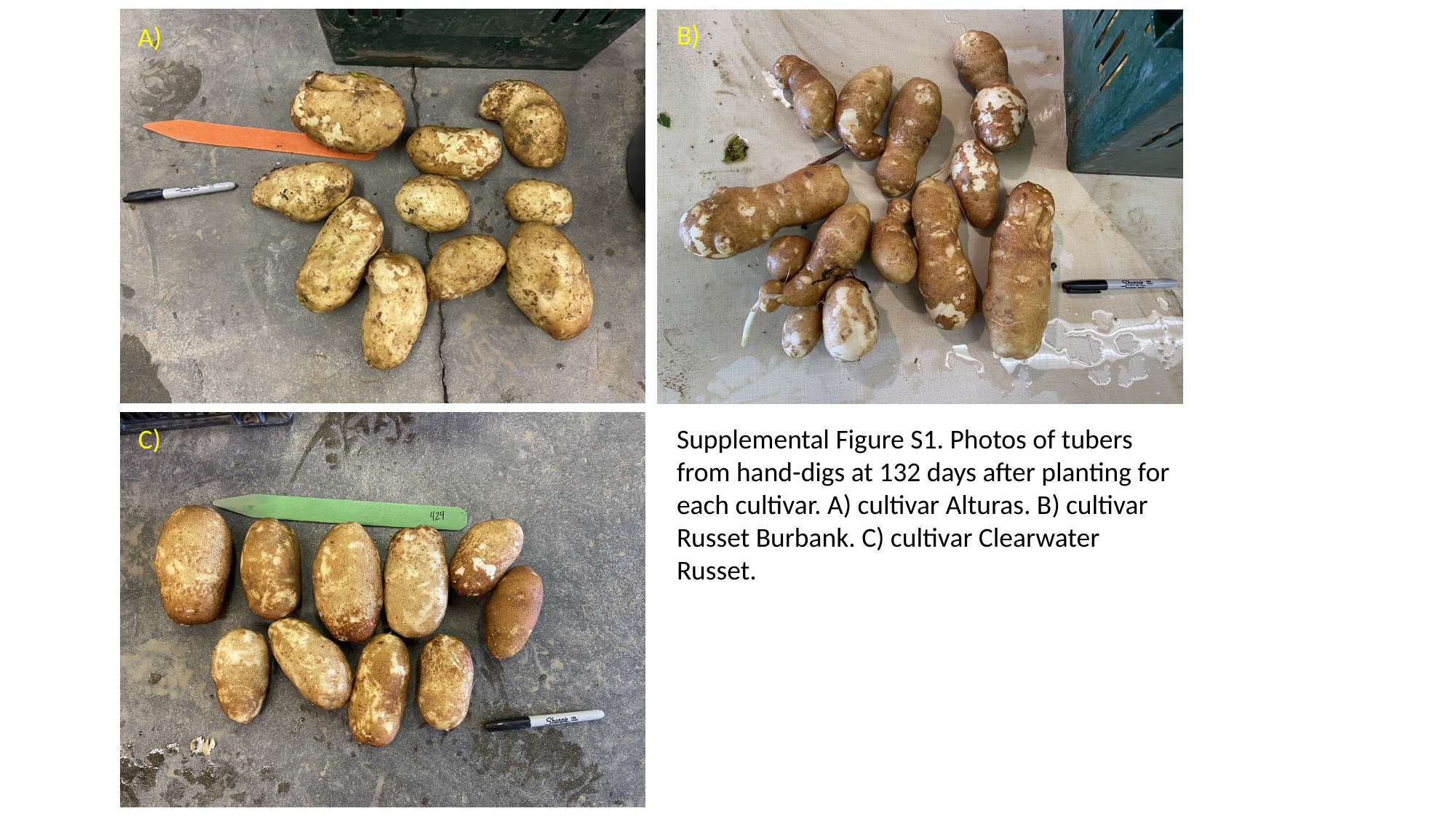

B)
A)
C)
Supplemental Figure S1. Photos of tubers from hand-digs at 132 days after planting for each cultivar. A) cultivar Alturas. B) cultivar Russet Burbank. C) cultivar Clearwater Russet.
